# Text Messages and Financial Incentives to Increase Physical Activity in Adolescents With Prediabetes and Type 2 Diabetes: Web-Based Group Interviews to Inform Intervention Design

**DOI:** 10.2196/33082

**Published:** 2022-04-06

**Authors:** Mary Ellen Vajravelu, Talia Alyssa Hitt, NaDea Mak, Aliya Edwards, Jonathan Mitchell, Lisa Schwartz, Andrea Kelly, Sandra Amaral

**Affiliations:** 1 Division of Pediatric Endocrinology, Diabetes, and Metabolism Department of Pediatrics University of Pittsburgh School of Medicine Pittsburgh, PA United States; 2 Center for Pediatric Research in Obesity and Metabolism University of Pittsburgh School of Medicine Pittsburgh, PA United States; 3 Division of Pediatric Endocrinology Johns Hopkins University School of Medicine Baltimore, MD United States; 4 Division of Endocrinology & Diabetes The Children’s Hospital of Philadelphia Philadelphia, PA United States; 5 Division of Nephrology The Children’s Hospital of Philadelphia Philadelphia, PA United States; 6 Division of Gastroenterology, Hepatology and Nutrition The Children’s Hospital of Philadelphia Philadelphia, PA United States; 7 Department of Pediatrics Perelman School of Medicine University of Pennsylvania Philadelphia, PA United States; 8 Center for Childhood Cancer Research Division of Oncology The Children's Hospital of Philadelphia Philadelphia, PA United States

**Keywords:** diabetes mellitus type 2, adolescent, young adult, text messaging, physical activity, motivation, mobile phone

## Abstract

**Background:**

Physical activity is a major component of treatment for adolescents with obesity and prediabetes or type 2 diabetes; however, sedentary behavior remains pervasive. An SMS text message–based intervention paired with financial incentives may be an effective way to promote physical activity in this population.

**Objective:**

This study aims to obtain end-user feedback on SMS text message content and assess the acceptability of a planned SMS text messaging intervention with financial incentives to motivate youth with prediabetes or type 2 diabetes to increase physical activity.

**Methods:**

Adolescents with overweight or obesity and prediabetes or type 2 diabetes who attended a large academic pediatric endocrinology clinic were recruited to participate in group interviews (2-4/group) via videoconferencing. Participants were asked to share their thoughts on the use of SMS text messages and financial incentives to remind and motivate them to be more physically active. They rated and provided feedback on specific messages to be used in clinical trials. Participants were also asked about their personal experience with rewards to motivate behavior change and their anticipated reactions to rewards provided for goal attainment (gain-framing) versus those provided and then taken away if a goal was not met (loss-framing). The interviews were conducted by 2 trained interviewers and a note-taker. Content analysis was used to explore themes.

**Results:**

Group interviews were completed with 20 participants (11/20, 55% women; 15/20, 75% with type 2 diabetes; 5/20, 25% with prediabetes) with a mean age of 15 (SD 1; range 12-18) years and a mean BMI of 41 (SD 5) kg/m^2^ (all >95th percentile for age and sex). Most participants were non-Hispanic Black (14/20, 70%) and 10% (2/20) were Hispanics. Participants frequently cited near-continuous smartphone use and agreed that SMS text messages would serve as good reminders to be physically active, but the consensus about the need for short messages was strong. Favorable content included references to what they were likely to be doing when messages were sent (eg, homework or watching television) and messages that were upbeat or informative. Specific physical activity suggestions were rated favorably. Attitudes toward financial incentives varied, with differing opinions about whether loss-framed incentives would be motivating or discouraging. Many participants highlighted the role of intrinsic, rather than extrinsic, motivation in achieving and sustaining behavior change.

**Conclusions:**

The engagement of adolescents with obesity and diabetes or prediabetes allowed for the refinement of SMS text messages for our planned intervention, with an emphasis on short, upbeat, relatable, and informative messages. Although an SMS text messaging intervention using financial incentives to motivate youth with prediabetes or type 2 diabetes to be more physically active is theoretically acceptable, the impact on actual activity levels in this population requires prospective evaluation in a clinical trial.

## Introduction

### Background

Once a disease of adulthood, type 2 diabetes is now becoming increasingly common among youth [1]. Among individuals with youth-onset type 2 diabetes, severe diabetes-related complications such as retinopathy, neuropathy, and nephropathy begin to emerge within 10 to 20 years of diagnosis and contribute to a 15-year reduction in life expectancy [2,3]. The increasing incidence is driven largely by obesity, which is present in 21% of persons aged 12 to 19 years in the United States [4] and quadruples the risk of youth-onset type 2 diabetes [5]. Globally, nearly one-fifth of children and adolescents aged 5 to 19 years old were overweight or obese in 2016, representing a more than 4-fold increase over 4 decades [6]. Prediabetes, which increases the risk of developing type 2 diabetes [7,8], is already present in approximately 20% to 30% of adolescents with obesity [9,10]. A healthy lifestyle, including adequate physical activity, plays a central role in the prevention and treatment of diabetes. Unfortunately, adolescents with type 2 diabetes typically do not reach the recommended duration of 60 minutes per day of moderate to vigorous physical activity [11], averaging only 8 (girls aged 15-18 years) to 26 (boys aged 10-14 years) minutes daily [12]. To reduce the risk of poor outcomes, effective methods to increase physical activity in youth with prediabetes and type 2 diabetes are needed.

Approximately 95% of teenagers in the United States now report ownership or access to a smartphone [13]. Smartphone ownership is also common throughout the world, although it is more prevalent in advanced economies (median 76% ownership of a smartphone; 17% of mobile phones that are not smartphones) than in emerging economies (median 45% ownership of a smartphone; 33% of mobile phones that are not smartphones). Notably, smartphone ownership is more common among younger individuals [14]. With such a high penetrance of smartphone and mobile phone ownership, one strategy to engage and motivate adolescents to be more physically active is via a text message–based intervention. Text message–based physical activity interventions have been studied in adolescents with overweight and obesity [15]; however, explicit focus on youth with prediabetes and type 2 diabetes has been limited [16]. To promote engagement and retention in a text message–based lifestyle intervention, however, it is critical to elicit adolescent viewpoints as part of intervention development [17,18]. This engagement is particularly needed for youth with obesity-related complications such as type 2 diabetes or prediabetes, in whom the perceived risk of poor health may impact responses to message tone and content.

Another potentially effective strategy to motivate youth to be more physically active is the provision of financial incentives. In adults, incentives framed as losses (upfront endowment with losses applied for failure to meet a goal) rather than gains (money earned upon completion of a goal) are more effective at inducing physical activity-related behavior change [19]. Financial incentives may also help counteract the rapid habituation of text message–based interventions that have been demonstrated in adolescents and young adults [20]. However, to our knowledge, the effectiveness of, or perspectives on, different financial incentive strategies to motivate adolescents to engage in physical activity have not been explored. In the Behavioral Economics for Activity Motivation (BEAM) trial (NCT04874415), a mobile health (mHealth)-based optimization trial, we are studying text messaging, loss-framed financial incentives, and gain-framed financial incentives as candidate components in a factorial experiment with youth with overweight or obesity and prediabetes or type 2 diabetes, with the aim of increasing time spent in moderate to vigorous physical activity.

### Objectives

To prepare for the trial, we conducted group interviews with adolescents from the target population. We sought insights into message content and financial incentive strategies, including financial incentive preferences and the anticipated impact on one’s motivation to be physically active.

## Methods

### Participants

A convenience sample of individuals from the target population of the BEAM trial, adolescents aged 13 to 18 years with overweight or obesity (BMI ≥85th percentile for age and sex) and prediabetes (hemoglobin A_1c_ 5.7%-6.4%, fasting glucose 100-125 mg/dL, and 2-hour oral glucose tolerance test glucose 140-199 mg/dL) or type 2 diabetes (hemoglobin A_1c_≥6.5%, fasting glucose ≥125 mg/dL, and 2-hour oral glucose tolerance test glucose ≥200 mg/dL and negative diabetes autoantibodies) were recruited from the pediatric endocrinology clinic at the Children’s Hospital of Philadelphia, a large academic children’s hospital in Philadelphia, Pennsylvania, United States, from December 2020 to April 2021. Potentially eligible participants were approached about the study at clinic visits or by phone within 2 weeks after a clinic visit by the study research coordinator or principal investigator. Individuals with limited English proficiency were excluded from this study. Eligible individuals were invited to participate in a 1-hour group interview. Electronic consent and assent (for participants aged <18 years) were obtained via REDCap (Research Electronic Data Capture; Vanderbilt University). Group interviews were roughly segmented by age when possible (13 to 15 or 16 to 18 years) to promote more open dialogue among similarly aged peers but not by other demographic or clinical characteristics. Participants were compensated with US $30 for their time. Limited medical record reviews to include demographic information and pertinent medical history were performed by the study team with the permission of the participants.

### Ethics Approval

Ethics approval was obtained from the Children’s Hospital of Philadelphia Institutional Review Board (IRB 20-017554).

### Message Development

The text messages were developed by the investigators with the assistance of one high-school student (female, aged 17 years) and one college student (female, aged 20 years). Both students became involved after approaching the primary investigator regarding temporary research opportunities in pediatrics, independent of any school-required research experience. Messages were designed with the target population in mind; limited financial resources and transportation barriers are common, so physical activity suggestions included only those that were free or low-cost, as well as many that could be performed at home. Initial message development was guided by common barriers to being more physically active, including feeling too tired, lack of interest, lack of peer or family support, lack of equipment, lack of space indoors or outdoors, lack of motivation, preference to avoid sweating, embarrassment, lack of confidence in skills, and lack of knowledge about how to do so [21]. Messages were crafted to have different tones, including informative, encouraging, or funny, to enhance variety and improve interest and engagement. Messages often included emojis, and some included graphic interchange formats (GIFs). The initial messages were reviewed and edited by the study team for clarity and their potential to engage end users. A total of 84 messages (1 per day for the 12-week intervention) were created.

### Web-Based Group Interviews

Group interviews were chosen rather than individual interviews to promote an atmosphere of engagement and the sharing of ideas among peers. Smaller group interviews (goal of 2 to 6 participants) were chosen over larger group interviews or focus groups (the original goal of 7 to 10 participants) because of the virtual nature of the interviews, which was necessitated by the COVID-19 pandemic. Although the original study design included the use of focus groups, the richness of the data gathered from focus groups is heavily dependent on the ability to promote and maintain dynamic group discussions. It is possible to conduct focus groups in a virtual setting, but potential challenges include technological difficulties and the inability to control each individual’s environment, which may lead to distractions and interruptions [22]. Therefore, we chose group interviews, in which the primary objective of the interviewer questions was to obtain individual responses rather than to stimulate group discussion as a method of information gathering [23]. Interviews were conducted by 2 primary interviewers (NM and AE), clinical research coordinators who identify as Black women in their 20s to 30s. NM had previous training and experience as a focus group facilitator and served as the primary interviewer. AE also participated in the recruitment of participants. MEV, the principal investigator, is a female pediatric endocrinologist who provides clinical care and conducts research with youth with prediabetes and type 2 diabetes; she assisted with interviews and identified the participants as White and Hispanic.

A semistructured interview guide was designed to elicit open-ended feedback on text messages for physical activity motivation (including acceptability, practical considerations, and preferred or nonpreferred content), experience with and motivation for behavior change, and experience with and attitudes toward the use of financial incentives for behavior change. The interview guides were piloted with nonmedically trained adults and revised as needed for clarity. The interviews were rehearsed by the interviewers with oversight by the study principal investigator (MEV), who had formal training in qualitative research methods. Interviewers practiced neutral, nondirective responses to participant answers and the use of open-ended probing questions to elicit more detailed responses. Before the start of the interview, interviewers informed participants that there were no correct answers and that all feedback, including critical feedback, was welcomed.

In addition to specific questions, 8 messages were reviewed during each interview. The number of messages was restricted to 8 to optimize the amount of time available for discussion, with the goal of understanding what aspects of the message were engaging or not rather than simply rating the specific message. Messages were selected to include a variety of barriers addressed and tones used (informative, encouraging, or funny) such that all combinations were tested at least once throughout the study. The messages were not repeated across groups; in total, 64 representative messages were reviewed. The remaining 20 messages were not substantially different in tone or content and thus were not reviewed by the study participants. During the interviews, message content was shared with participants via the videoconferencing platform’s *screen* *share* function and read aloud. Participants were asked to rate the messages as *great*, *OK*, or *bad*; these specific rating words were chosen to maximize the ability to identify message *outliers*—those that truly resonated (*great*) and those that were strongly disliked (*bad*)—so that themes relating to message success or failure could more easily be identified. Participants were invited to type their initial ratings in the platform’s chat or respond aloud. After all initial ratings were shared, the participants were asked to explain their ratings, including why they liked or disliked the message and how it could be improved.

All interviews were conducted via the videoconferencing platform BlueJeans (Verizon), accessed by participants using their preferred personal internet-connected device (smartphone or computer). Participants were asked to keep their camera on if comfortable doing so and to remain unmuted in a quiet setting if possible. If participants wrote in the chat rather than speaking, the interviewers reread the typed content, stating which participants responded.

### Analysis

Participant characteristics were presented using summary statistics, including means and SDs for continuous variables and proportions for categorical variables. All interviews were recorded securely on a videoconferencing platform, and audio was transcribed using a professional transcription service. Transcripts were reviewed by the study staff, corrected as needed, and supplemented by observer notes. Transcripts were deidentified before analysis. Content analysis was conducted to explore these themes. An a priori set of codes was created based on the semistructured interview guide; for example, *Text messages acceptability* and *Text messages: preferred content*. Next, two of the study team members with experience and training in qualitative study methods (TAH and MEV) reviewed the transcripts, identified emergent themes, and then refined the initial codebook. For example, an additional code for *pandemic* was considered owing to the frequent mention of how the COVID-19 pandemic impacted behavior and motivation but ultimately not included as an independent code but rather incorporated as a subcode to motivation for behavior change. After independently coding the 3 transcripts, the 2 reviewers compared the coding and clarified the codebook as needed. NVivo (version 12; QSR International) was used for qualitative analysis.

## Results

### Participant and Group Interview Characteristics

Participants (N=20) had a mean age of 15.7 (SD 1.3) years; 75% (15/20) had type 2 diabetes, and 25% (5/20) had prediabetes. The majority (14/20, 70%) of participants were non-Hispanic Black, and the remainder were non-Hispanic White (3/20, 15%), Asian (2/20, 10%), or mixed race (1/20, 5%); of the 20 participants, 2 (10%) participants were of Hispanic ethnicity. Among the participants with diabetes, 53% (8/15) were prescribed insulin, 93% (14/15) were prescribed metformin, and 13% (2/15) were prescribed liraglutide. The median most recent hemoglobin A_1c_ level was 6.1% (IQR 5.9%-6.2%) among participants with prediabetes and 7.3% (IQR 6.5%-10.4%) among participants with diabetes (Table 1).

In all, 8 group interviews, each consisting of 2-4 adolescents, were conducted from December 2020 to April 2021. Each group interview lasted for approximately 30 to 45 minutes. The participants had variable degrees of engagement, with many leaving their cameras off. However, all (20/20, 100%) participants responded to the entire set of interview questions and rated all text messages per session. Many used the chat feature in the videoconferencing platform to respond but spoke aloud when asked to do so.

**Table 1:** Participant characteristics.

Category		All participants (N=20)	Participants with prediabetes (n=5)	Participants with type 2 diabetes (n=15)
**Sex, n (%)**				
	Female	11 (55)	3 (60)	8 (53)
	Male	9 (45)	2 (40)	7 (47)
**Race, n (%)**				
	Black	14 (70)	3 (60)	11 (73)
	White	3 (15)	0 (0)	3 (20)
	Asian	2 (10)	1 (20)	1 (7)
	Mixed	1 (5)	1 (20)	0 (0)
**Ethnicity, n (%)**				
	Non-Hispanic	18 (90)	5 (100)	13 (87)
	Hispanic	2 (10)	0 (0)	2 (13)
Age (years), mean (SD)		15.7 (1.3)	15.6 (1.5)	15.7 (1.3)
BMI (kg/m^2^), mean (SD)		40.5 (5.5)	40.5 (6.7)	40.5 (5.3)
**Insulin use, n (%)**				
	No	12 (60)	5 (100)	7 (47)
	Yes	8 (40)	0 (0)	8 (53)
Hemoglobin A_1c_ (%), median (IQR)		6.6 (6.1-9.1)	6.1 (5.9-6.2)	7.3 (6.5-10.4)

### Themes

#### Overview

Thematic saturation was achieved with no new themes generated from later interview groups. Instead, previously identified themes were also present in the later groups. The interrater reliability of the 8 transcripts was high (*κ*=0.92). Several themes and subthemes emerged from focus group discussions that pertained to the development of text messaging content as well as the use of financial incentives in an intervention designed to motivate youth with overweight or obesity and prediabetes or type 2 diabetes to be more physically active.

#### Theme 1: Near-Continuous Use of Smartphones and Acceptability of Text Message Reminders

All but one (19/20, 95%) participant had smartphones and felt that text messaging was a good way to remind teens to be more active:

We always on our phone, so we gonna see it [and] texting is a really good way to sort of spread words. I mean, I know that I, personally, spend probably way too much time on my phone.

Participant 19, group 8; female, aged 14 years

The interruption via text message was felt to be a useful prompt to move more:

I feel like if you - if we got manual reminders, it could implant something in our head, and like an idea in our head to do it instead of forgetting about it throughout the day.

Participant 19, group 8; female, aged 14 years

Participants varied in the acceptable frequency of text message reminders, with a participant suggesting that messages as frequently as once per hour would be appropriate. However, most participants preferred 1-2 messages per day, either at noon or after school. They emphasized the need to know from whom the text was sent, so they did not mistake it for spam. Most felt that they used their phone more during the summer but had it with them at nearly all times, regardless of the time of year or day of the week.

#### Theme 2: Text Messages Should Be Short, Upbeat, Informative, and Relatable

Short and upbeat messages were strongly preferred. Table 2 highlights representative text messages and participant responses. Participants reported that they would most likely read and enjoy messages that were 1 sentence or phrase long and noted that they may not open the message to read beyond the message preview. Regardless of the originally intended tone (informative, encouraging, or funny), messages that had *upbeat* characteristics such as exclamation points, smiley faces, or encouraging words were felt to be most motivational. However, even if brief and encouraging, those stating a generally known fact (eg, *Exercise helps your body and your brain*) resulted in mixed responses. Importantly, a participant highlighted the need for caution when discussing self-esteem. Reflecting on the message *Did you know that being physically active can improve your self-esteem? Why not start right now? You got this 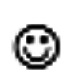,* she stated that this comment would cause her to question her own self-esteem:

While self-esteem is a great thing to focus on and have, I think if you point it out, it makes us feel a little bit insecure...If I got this sent to me and I was like - and I looked at it, I’d be like do I have low self-esteem?

Participant 1, group 1; female, aged 16 years

Participants reported satisfaction with texts that reported facts or specific suggestions on how to overcome barriers: *Feel like you’re tight on time? Maybe it’s in your head. A brisk walk actually changes the chemicals in your brain to make you feel more relaxed and less anxious* 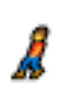 was felt to explain a bit of the science and to be memorable.

Another informative message (*Physical activity that gets your heart rate up releases endorphins that raise your energy level. Ready for your energy boost?*) 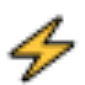 was appreciated for providing a specific reason for exercising. Messages that gave examples of how to overcome specific barriers were also well-rated. For example, in response to a message that identified the common barrier of feeling embarrassed to exercise in front of others and suggested a solution (*It is completely normal to feel embarrassed to work out around others. Don’t let that stop you. Exercise by yourself or with people you’re comfortable with*), a participant rated the message highly:

...It acknowledges a common feeling that a lot of people feel when they’re working out around other people. And then on top of that, it gives a suggestion as to how you can fix that feeling...

Participant 20, group 8; male, aged 14 years

There was agreement on the utility of sharing ideas to be physically active, but the preferred activities to suggest varied by participant. Several noted that they did not like sports, that “yoga sounds hard,” exercise classes sounded like school (“Why must I have a class? Why can’t we just go outside and just do whatever? Why does it have to be a class?” [Participant 13, group 6; male, aged 15 years]), and not everyone has room to or enjoys dancing at home. Suggestions about how to be physically active at home were appreciated, but participants differed in the perceived utility of including a link to a video or description of activities, with many stating that they would click the link, but others suggesting that clicking the link is “just kind of another thing to do.”

Messages that mentioned school or sitting on the couch and watching television were highly relatable. A message that emphasized that being active can help with school performance resonated with a participant:

I like how it ties in the school thing. I know that I would like to do better in school. And I think that it’s the same for a lot of people my age. So, I think that this one could really capture a lot of people.

Participant 10, group 4; male, aged 18 years

**Table 2:** Example text messages, tone, and feedback.

Message	Tone	Representative quotes
Treadmills get you nowhere 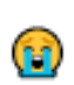 . But seriously, walking is great exercise!	Funny	“I like how it’s kind of not comedic, but it’s kind of like the exclamation point adds like a sense of - yeah, like upbeat...I like how the other one had like a fact because fun facts interest me and will grab my attention more” (participant 12, group 5; female, aged 13 years).
No matter how slowly you walk or jog, you are still lapping everybody on the couch 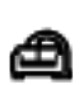 !	Funny	“I said great because think about like if we were all sitting on the couch or whatever being lazy, then someone got this text message, they could maybe motivate everybody else, like let’s just go on a walk together” (participant 11, group 5; female, aged 16 years). “...When you get this, let’s say you’re watching a movie. Everybody’s on the couch and eating chips or whatever, and you see this emoji or you see this message. It’s like a reminder to like go out, and it’s like motivational” (participant 12, group 5; female aged 13 years).
Sometimes large gyms are not for everyone; make use of smaller spaces such as a bedroom or a kitchen to fit in a quick work out. At least it will smell better 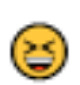 !	Funny	“[It’s] ok—I would change it to make use of the space you are most comfortable in” (participant 4, group 2; female, aged 14 years).
Exercise helps your body and your brain, so get to it 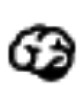 !	Informative	“I heard this before still, but I think it’s like a short and simple nice quote to educate people on and to send a quick reminder because like that exercising can put you in a better mood and stuff like that” (participant 11, group 5; female, aged 16 years). “And I like that it’s short, but it doesn’t give me motivation or anything. It’s just like another text that would be sent, read, and not replied to, and wouldn’t have any affect in my life” (participant 12, group 5; female, aged 13 years).
Feel like you are tight on time? Maybe it’s in your head 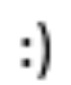 . A brisk walk actually changes the chemicals in your brain to make you feel more relaxed and less anxious 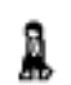 .	Informative	“I feel like this one’s great as well because if you’re ever feeling anxious, you can go back to this in your brain and remember that one time you got a text message telling you how to fix it. So I think this is good for any situation where you’re ever feeling anxiety” (participant 19, group 8; female, aged 14 years). “I think this one’s great because it explains it. It explains a bit of the science” (participant 20, group 8; male, aged 14 years).
*A* for effort: being active can help you do better in school 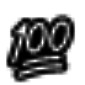 .	Informative	“I like how it ties in the school thing. I know that I would like to do better in school. And I think that it’s the same for a lot of people my age. So, I think that this one could really capture a lot of people” (participant 8, group 4; female, aged 14 years).
Improving your fitness is a slow process, but quitting will not speed it up! Keep up the great work!	Encouraging	“I know a lot of people do better with motivation, so it’ll help motivate you during that day. Whatever you’re doing, you see this and you’re like okay. Somebody believes in me...” (participant 12, group 5; female, aged 13 years).
Rise and shine 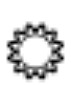 . Do something today that your future self will thank you for. Get yourself pumped up!	Encouraging	“I like this one. It’s motivational and I think I’d like reading that in the morning...Yeah, this is a great thing to tell yourself or wake up to reading every day, in my opinion” (participant 18, group 7; female, aged 16 years). “Yeah, I like that one, too. It’s very motivational” (participant 16, group 7; male, aged 15 years).
Exercising with others can make it fun and help motivate you. Who will be your work out buddy today 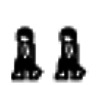 ?	Encouraging	“...There’s really nothing outstanding about it. I do like how it sort of – it tells you to get with a friend and do that sort of thing. I like that idea” (participant 10, group 4; male, aged 18 years). “It’s good, but I prefer to work out by myself” (participant 9, group 4; female, aged 17 years).

Participants imagined themselves receiving messages while sitting on the couch and *being lazy*, then felt motivated to stand up and walk around or even exercise during commercial breaks. A participant reflected as follows:

...Think about like if we were all sitting on the couch or whatever being lazy, then someone got this text message, they could maybe motivate everybody else, like let’s just go on a walk together.

Participant 11, group 5; female, aged 16 years

Another participant agreed:

I think that when you get this, let’s say, you’re watching a movie. Everybody’s on the couch and eating chips or whatever, and you see this emoji or you see this message. It’s like a reminder to like go out, and it’s like motivational...

Participant 12, group 5; female, aged 13 years

#### Theme 3: Extrinsic Versus Intrinsic Motivation to Change Behavior

Participants frequently reported that they had a goal to exercise more, but they differed in their sources of motivation or encouragement to do so. Parents and physicians were often cited as the person setting physical activity goals for participants, and frequent reminders were felt to be particularly frustrating. A participant noted that “a lot of adults tell kids to exercise and that they need to get out more” by quoting the following:

And I get it. Not helpful when I’m in the middle of doing something, and then someone comes into my room and just like get outside. I’m just like why? I also tend to not exercise when there is no immediate goal...It needs to have a sensical purpose.

Participant 1, group 1; female, aged 16 years

Another participant expressed frustration about being reminded to exercise:

When I’m told over and over again to exercise and what will happen if I don’t, it’s – because I make sure that I’m exercising as frequent as possible...So when people...say why aren’t you exercising or you need to exercise more, it can get annoying and it can make me not want to do it.

Participant 12, group 5; female, aged 13 years

A participant emphasized the value of a parent taking time to show concern when encouraging him to be more physically active:

It’s like she actually sat down with me, instead of just telling me. She actually sat down. Actually, had a nice conversation about it, instead of just coming to my door and telling me what to do.

Participant 8, group 2; male, aged 14 years

Personal and family history of type 2 diabetes were major motivators for several participants to engage in a healthy lifestyle. A participant shared the following:

...My motivation is so I can get off of these pills I take, the metformin and whatnot...I’ve been doing absolutely everything I can because I have family members who have the severe type two diabetes, and I don’t want that to be me...I don’t want to lose my life due to health issues...I don’t want to pass away when I’m 40 because I have serious health issues, so I want to live my life the right way and be normal for once.

Participant 15, group 7; male, aged 16 years

Another reflected by quoting the following:

Saw kind of the direction that my health was going in, and it was kind of like concerning, so I wanted to turn that around so that before it got too permanent or too bad or anything like that, so that was something that motivated me for sure.

Participant 19, group 8; female, aged 14 years

#### Theme 4: Effect of Financial Incentives May Depend on Intrinsic Motivation

Nearly all participants reported that their parents had offered rewards for behavior change, most commonly money or a desired item such as a video game or clothes. However, the perceived effectiveness and acceptability of this approach varied. Some reported that a monetary reward or desired object “kind of pushes you more and gives you a good boost of energy and motivates you a lot” (participant 11, group 5; female, aged 16 years) and that “I like the idea of getting something out of doing things” (participant 10, group 4; male, aged 18 years). On the other hand, another participant questioned the use of financial incentives when discussing her experience with an allowance: *“...When I started getting - becoming like older and more slightly anarchist, it was a bit of why am I doing this for capital reasons? I don’t like this*” (participant 1, group 11; female, aged 16 years).

The perceived importance of the underlying behavior to change was a major factor in the persistence of behavior change. Participants reported feeling motivated to continue the behavior if habits were “*good*” for themselves or others, but if “[T]hey weren’t helping people that much...I really saw no point in doing it” (participant 10, group 4; male, aged 18 years). Being physically active was categorized by some respondents as “just the right thing to do,” which negated the use of financial incentives. In addition, the end goal was emphasized:

It’s a good chance that you’re going to meet your goal when you have the motivation, like something that you’re working hard towards. Like for example, if I was - I know that I’m trying to work hard toward being healthy mentally and physically. Well, I know that we - all this hard work is eventually going to pay off. So, sometimes you’ve just got to - sometimes you ’ve just got to think and have that mindset. If I work hard, eventually it’s going to pay off in the end.

Participant 11, group 5; female, aged 16 years

Financial rewards were also seen as a way to overcome barriers even for goals that were previously intrinsically motivated:

...Last year before, pre-coronavirus, I was all-A student...And I didn’t need any money to motivate me...when I saw the honor roll in my hand, that was my motivation...But this year, I did not get all As and all Bs...as motivation [my dad] said at the end of the year, if I was to bring home an...all As and Bs honor roll, that I would get a shopping spree or money or stuff like that...but that is kind of the motivation that I do need to keep my grades up.

Participant 11, group 5; female, aged 16 years

However, participants identified that goals that are *only* extrinsically motivated by money may not be achieved when additional barriers arise:

...My parents used to give me a prize if I got like 100 on a test or something like that, and it always motivated me to study and do well in school...it pretty much worked until COVID hit because my grades started going down because I wasn’t that motivated.

Participant 12, group 5; female, aged 13 years

#### Theme 5: Potential Benefits and Drawbacks of Loss-Framed Incentives

When asked about loss-framed incentives, using either money or physical objects, participants gave examples of times that their parents had threatened or actually removed objects for poor behavior. Most participants expressed frustration, stating that this approach would make them “sad,” “mad,” and “defeated or upset” and that it would be “unmotivating.” However, some also acknowledged that this approach could be viewed as fair:

...I would understand why that got taken away from me. I know that it would be because I didn’t do whatever I was supposed to do, so I personally wouldn’t be angry because I know why that would get taken away from me.

Participant 11, group 5; female, aged 16 years

Another participant, speaking about her father’s threat of not allowing her to participate in an activity anymore if her grades fell, reflected the following:

...I felt neutral about the whole situation, but I was always - I think it helped me because I knew that that could be taken away because one thing about my dad’s ultimatums is that he means business...So I felt - not scared, but I was like okay. I know what I need to do.

Participant 12, group 5; female, ages 13 years

When evaluating preferences, perceived fairness, and effectiveness of loss- versus gain-framed incentives, opinions varied, with some participants reporting that either approach could work. Several participants acknowledged that the perception of loss would likely be highly motivating despite, or because of, the frustration it causes. A participant reflected the following:

...It is nice to have something earned but there should be some sort of consequence so you don’t become lazy. Earning something would be more successful because you can see your accomplishment.

Participant 6, group 2; female, aged 17 years

Some noted that they would feel highly motivated by the possibility of having something taken away, stating the following:

What motivates me more is like having something taken away, so then I would know that...I would get that back in the end after I work hard [and] the threat of having something taken away motivates me to do better, just so I could have it with me the whole time.

Participant 11, group 5; female, aged 16 years

However, participants felt that the effectiveness of loss- versus gain-framed rewards would depend on the reward itself:

It depends on what the reward is and it depends on what’s being taken away. So like if you took - personally, like if you took my [video game system] away, I’d be like yeah, I’d choose that one.

Participant 1, group 1; female, aged 16 years

Several others felt the following:

Positive reinforcement would be more effective and that the threat almost makes me want to revolt, because I don’t like threats...that promise, like that shopping spree promise, it motivates me. I’m like I’m almost there. I can see the finish line. But threats, no. So the promise of having a reward, because the threat is too much for me and it does not make me want to do my best. It makes me want to do worse just to prove that person - I don’t know, because it’s a really catty thing with me if I’m threatened...

Participant 12, group 5; female, aged 13 years

Importantly, although the behavior may change in the desired way because of the incentive, a participant questioned whether it would result in true motivation:

I think it’s the [threat of having something taken that works better] because you’re scared of doing it or not doing it, so you keep on doing it. And it’s not necessarily motivation, but it’s just like at the end of the day, you’re still doing it, so it’s good.

Participant 20, group 8; male, aged 14 years

### Content Revision

On the basis of the participant feedback, messages were refined by study team members for use in the BEAM trial. Those rated as *bad* by even a participant were edited to omit unfavorable content or discarded entirely if the overall concept was disfavored. For example, the message *Did you know that being physically active improve your self-esteem? Why not start right now? You got this* 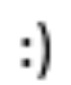 was omitted, and messages that were supportive of self-confidence and self-efficacy but that did not unintentionally imply that the individual had poor self-esteem, were substituted instead (*Ever feel like you’re in a tough situation? Going for a walk can help you prepare to face that problem and overcome it).* On the basis of the favorable response to messages referencing feeling lazy or describing how being physically active may help with school performance, more messages with these themes were created. Messages with mixed responses were edited based on specific negative feedback or included if responses were positive and neutral. Additional encouraging messages were created to address problem solving to overcome barriers to being more physically active and to remind the value of a support person to help achieve activity goals; for example, *Does your step count goal seem like a stretch? Break it up! Every minute counts!* and *Who will be your workout buddy today? Bring a friend or family member with you on a walk or to the gym (blame us if you need to!).* Messages were also shortened and adapted to fit the preferred timing in mid-day or afternoon (eg, *You have the power to start your day off strong! Wake up, have your favorite breakfast and go for a walk, run, or bike ride. Get moving and you’ll be ready to face the day* was changed to *Tomorrow, you have the power to start your day off strong! Plan to wake up, be active, and you’ll be ready to face the day),* and additional encouraging messages were created (eg, *Is working toward your fitness goal a bit challenging? That's great! The more challenging a task, the more rewarding it will be in the end!)* 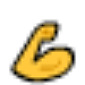. Additional specific lessons learned are summarized in Textbox 1.

**Lessons about the use of text messages and financial incentives for physical activity motivation among adolescents.**

**Acceptable message frequency**

- 1-2 texts per day

**Acceptable message timing**

- Mid-day or afternoon (after school)

**Ways to minimize the risk of messages being ignored**

- Save the study’s or team’s phone number in contacts so that it is not identified as spam
- Shorten messages or put most important text in beginning of message
- Use exclamation points and emojis

**Financial incentives for behavior change**

- Potentially motivating in theory
- Familiar concept to adolescents

**Loss-framing to promote behavior change**

- Acceptability divisive: motivating but possibly too frustrating

## Discussion

### Principal Findings

Through virtual group interviews of adolescents with obesity and prediabetes or type 2 diabetes, we obtained end-user feedback to refine a bank of text messages for an mHealth-based physical activity intervention and identified several themes related to the use of text messaging and financial incentives to motivate youth to be more physically active. The participants’ responses highlighted the challenge of developing message content that appeals to all end users [24], as well as the importance of keeping messages brief, upbeat, informative, and relatable. Adolescents’ familiarity with rewards as a means to encourage behavior change allowed a rich discussion of their anticipated responses to financial incentives for physical activity motivation.

Our approach to intervention development is *person-based*, in which qualitative research is used to inform design by gaining insight into the perspective and psychosocial context of individuals who will use the intervention [25]. This qualitative assessment will be used not only in the intervention design but also in the evaluation of the intervention. Stakeholder involvement is an essential component of digital health interventions [26], but to date, text messaging interventions promoting healthy lifestyle changes for adolescents with obesity have been heterogeneous, with a limited description of whether, and how, the intervention was co-designed with end users [15,27]. One recent exception is the TEXTBITES intervention, in which Partridge et al [27] used an iterative mixed methods approach to develop a text message program for Australian adolescents with obesity. We took a similar approach to develop a text-message–based physical activity intervention for adolescents with overweight or obesity and prediabetes or type 2 diabetes (BEAM trial). Like TEXTBITES, our population will also include adolescents with obesity, but as reflected in this study, a large proportion of participants in our trial will be youth of minority race or ethnicity and from disadvantaged socioeconomic backgrounds towing to the higher prevalence of type 2 diabetes and prediabetes in these populations [28,29].

The likelihood of limited financial resources or safe spaces to engage in physical activity shaped our physical activity suggestions, which were intentionally free or low-cost and included in-home options. In addition, our population also likely differs from otherwise healthy adolescents in terms of overall stress and depressive symptoms; youth with type 2 diabetes report high levels of life stressors, which correlate with poor psychosocial functioning and impaired treatment adherence [30]. Thus, for our ultimate goal of helping adolescents with prediabetes and type 2 diabetes to become more physically active, we worked to develop messages that were both encouraging and contained activity suggestions that were perceived as realistic and achievable.

Our finding that adolescents preferred messages that were brief, upbeat, and informative was similar to previous reports [18,31]. In their TXT Me! intervention, Thompson et al [18] used pedometers and stand-alone text messages grounded in concepts from the Self-Determination Theory, including autonomy, competence, and relatedness, to promote increased physical activity among adolescents [18]. They used web-based surveys and telephone interviews with 30 persons, aged 14 to 17 years, to assess the acceptability of pedometer use and daily texts to help achieve a step-count goal. Similar to our findings, the adolescents in the study by Thompson et al [32] favored short messages that were positive, used exclamation points, did not nag, and focused on facts. In a pilot study of their intervention [32], they demonstrated feasibility, with an enrollment of 160 adolescents and complete data available in 86% of cases. Notably, they found that participants who were excluded owing to insufficient pedometer use or missing data collection were more likely to be older and African American. Postintervention feedback included the suggestion that other types of physical activity should be promoted in addition to walking and that a step count goal should be set for participants rather than having the goal be self-selected. In total, 80% (16/20) of the adolescents enjoyed receiving daily texts for 12 weeks, and 75% (15/20) chose noon or afternoon as the best time to receive texts. Notably, participation was not based on body weight or health conditions, including obesity, type 2 diabetes, or prediabetes. In contrast, Woolford et al [31] explored attitudes and preferences regarding automated SMS text messages among adolescents with obesity as part of a multidisciplinary weight management program. The messages included an explicit focus on topics central to weight management. As demonstrated in our study and in TXT Me!, adolescents reported that an automated SMS text messaging strategy would be acceptable and preferred positive upbeat messages that included exclamation points [31]. Again, the study by Woolford et al [31] did not explicitly focus on adolescents with obesity-related complications such as prediabetes or type 2 diabetes.

To our knowledge, ours is the first study to evaluate the acceptability of a text message–based intervention to increase physical activity in adolescents with obesity and prediabetes or type 2 diabetes. Because of the critical importance of lifestyle changes in improving health outcomes, youth with prediabetes or type 2 diabetes are likely to have already been instructed by multiple health care providers and their caregivers to be more physically active. It is notable that despite this commonly heard advice, adolescents still reported that daily or twice-daily text messages encouraging and reminding them about physical activity would be acceptable and potentially motivating.

In addition to specific feedback about the reviewed text messages, participants shared valuable insights about experiences when encouraged to exercise by caregivers and health care providers. Their responses highlighted the fine line required when showing concern about an adolescent’s health and health behaviors. Although expressing genuine concern may be encouraging for some, repeated reminders may be perceived negatively by others. There is a danger that repeated text message reminders to *exercise* may also be perceived in this light. Our revised text message bank now places greater emphasis on being more *physically active* in any activity of their choice, rather than structured and repetitive *exercise*. Indeed, distinguishing between *exercise* (a planned, structured, purposeful, and repetitive behavior) and more general *physical activity* may help clarify expectations for patients, as increased nonexercise physical activity may be an acceptable goal for both patients and providers [33]. As part of the planned intervention, we will counsel participants’ caregivers about the benefit of showing support for the adolescent’s physical activity in ways that can be more naturally incorporated throughout their days, rather than insisting that the adolescent engages in narrowly defined exercise at specific times. The specific linkage of the health benefits of physical activity to diabetes-related outcomes may also prove motivational to youth already experiencing an obesity complication, driving intrinsic motivation rather than relying primarily on extrinsic motivation. As described by the health belief model, the perception of health vulnerability associated with prediabetes or type 2 diabetes may serve as an additional motivation to act [34] on healthy lifestyle changes. This concept was suggested by some youth who referred to their health as a primary motivator to increase physical activity, but again, health care providers should balance using health as a motivator while not inducing shame or stigma that is commonly associated with obesity and type 2 diabetes [35].

Because of the challenges of maintaining engagement in mHealth and physical activity interventions as well as motivating adolescents to be more physically active, we also tested the impact of different financial incentive strategies in our BEAM trials. Specifically, we evaluated loss- versus gain-framed financial incentives to achieve step-count goals. Although the comparative efficacy of these financial incentive approaches has been evaluated in physical activity interventions in adults, it has not been assessed in youth [36], who may differ in their responses to the negativity of a loss-framed incentive. Among adolescents with type 1 diabetes, financial incentives appear to be an acceptable approach for promoting self-monitoring behaviors [37]. However, the acceptability of loss-framed incentives has not been explored. When evaluated in a clinical trial, loss-framed financial incentives led to more frequent blood glucose monitoring among adolescents with sub optimally controlled type 1 diabetes [38]. Participants in that trial endorsed the feasibility of daily financial incentives to motivate behavior change, with some reporting feeling motivated by the loss of money they believed was already their own. Notably, however, the increase in self-monitoring behaviors was transient, and the effect was extinguished after financial incentives were stopped. The possibility of financial incentives to crowd out intrinsic motivation has led to the concern that financial incentives may do more harm than good [39]; fortunately or unfortunately, this is less of a concern for health behaviors, many of which are associated with low baseline intrinsic motivation [40]. Our exploration of attitudes toward gain- and loss-framed incentives suggested that the acceptability of loss-framed incentives is mixed but that some adolescents may find them highly motivating. This study was not designed to quantify the differences in the anticipated motivation or acceptability of different financial incentive approaches. However, in the BEAM trial, we explore the heterogeneity of the treatment effect of the incentives in an effort to identify characteristics that predicted objective responsiveness to different financial incentive approaches.

Although not initially planned, owing to the COVID-19 pandemic, we conducted group interviews using a virtual format using videoconferencing technology. Virtual interviews had several benefits: (1) ease of recruitment, particularly for adolescents with financial or transportation barriers; (2) efficient and high-quality audio recording via the platform; and (3) availability of the chat feature, which allowed participants who may not otherwise have interrupted someone speaking to share their thoughts and engage in the discussion. The virtual format allowed participants to mute video or audio, which was done at times the strain discussion progressed. However, the ability to retain control of their privacy, which is not practical in most traditional focus groups [41], may have encouraged participants to share more freely than they would have in an in-person setting. Technical challenges occasionally arose, including poor audio quality related to internet connectivity. Overall, the virtual format appears to be a promising way to engage adolescents in qualitative research in a way that minimizes participant burden while facilitating recruitment. Our successful engagement of participants in a remote format also provides further evidence of the feasibility and acceptability of mHealth interventions targeting adolescents [42,43].

### Limitations

Attitudes and perspectives about text message content and financial incentives may vary across populations; therefore, our findings may not be generalizable to other adolescent subgroups, such as those with obesity but without comorbidities, or to individuals of different age groups or at different neurodevelopmental stages [36]. However, our participants represent a diverse group whose perspectives are not often elicited or reflected in research settings and represent the epidemiology of youth with prediabetes and type 2 diabetes, who are disproportionately from minority backgrounds [29]. As with any group interview or focus group study, particularly those including adolescents, social desirability bias may have influenced the responses [44]. We did not use single-sex groups, as has been recommended when conducting qualitative research with groups of unfamiliar adolescents [44]. However, our small group sizes may have limited the potential discomfort or embarrassment of offering differing opinions. Because the BEAM trial uses text messages in English, we did not include participants with limited English proficiency. We acknowledge that this has the potential to limit the intervention’s reach among Hispanic youth, who have a higher prevalence of type 2 diabetes than non-Hispanic White youth [29]. Importantly, to adapt text message interventions for individuals with limited English proficiency, it is critical to embark on a *transcreation* process, in which the text messages are not simply translated but adapted in a way that is linguistically and culturally appropriate [45]. Thus, additional qualitative research is required to evaluate the acceptability and appropriateness of the adapted intervention.

The focus of this study was on the perspectives and preferences regarding text messages and financial incentives to motivate behavior change, which may not perfectly correlate with objectively measured behavior change; objective measurement will occur as part of the BEAM trial. Because of our study’s sample size, we were unable to reliably characterize differences in attitudes and perceptions across patient characteristics, such as age, gender, or prediabetes versus diabetes; however, we will do so when evaluating the acceptability of the intervention in the BEAM trial. The use of virtual interviews made engaging participants challenging at times; this limited engagement often contributed to more limited discussion than may have occurred in face-to-face interviews and thus to the shorter actual duration of interviews than originally planned (30-45 vs 60 minutes). Finally, although participants reported that they found even frequent text messaging acceptable, their actual behavior may differ, as additional interruptions from push notifications and text messages from friends also occur. The BEAM trial will also evaluate the impact of message frequency.

### Conclusions

In summary, among the participants in our study, all adolescents with obesity and prediabetes or type 2 diabetes preferred text messages that were short, upbeat, informative, and relatable. Through virtual group interviews, participants’ feedback allowed for the creation of an end-user–refined bank of text messages that will be used in an mHealth physical activity intervention targeting the same population (BEAM trial). The perceived acceptability of loss-framed incentives was mixed, but the effect of incentive framing will be objectively measured in the BEAM trial. Overall, the ability to elicit end-user feedback increased the likelihood of acceptability of our intervention among the target population, and we will continue to engage participants throughout the trial to further refine our approach to intervention development.

## Abbreviations

BEAM: Behavioral Economics for Activity Motivation
GIF: graphic interchange format
mHealth: mobile health
NIH: National Institutes of Health
REDCap: Research Electronic Data Capture
